# Performance of whole-genome promoter nucleosome profiling of maternal plasma cell-free DNA for prenatal noninvasive prediction of fetal macrosomia: a retrospective nested case-control study in mainland China

**DOI:** 10.1186/s12884-022-05027-w

**Published:** 2022-09-10

**Authors:** Qianwen Lu, Zhiwei Guo, Jun Zhang, Ke Wang, Qi Tian, Siping Liu, Kun Li, Cailing Xu, Caimin Li, Zenglu Lv, Zhigang Zhang, Xuexi Yang, Fang Yang

**Affiliations:** 1 Department of Fetal Medicine and Prenatal Diagnosis, Zhujiang Hospital, Southern Medical University, Guangzhou, 510280 China; 2 Department of Obstetrics and Gynecology, Nanfang Hospital, Southern Medical University, Guangzhou, 510515 China; 3 Institute of Antibody Engineering, School of Laboratory Medical and Biotechnology, Southern Medical University, Guangzhou, 510515 China; 4 Department of Obstetrics and Gynecology, The Third Affiliated Hospital of Sun Yat-sen University, Guangzhou, 510630 China; 5 Department of Pathology, Cangzhou People’s Hospital, Cangzhou, 061000 China

**Keywords:** Fetal macrosomia, Classifier, Cell-free DNA, Low-coverage whole-genome promoter profiling, Noninvasive prediction

## Abstract

**Background:**

Fetal macrosomia is common occurrence in pregnancy, which is associated with several adverse prognosis both of maternal and neonatal. While, the accuracy of prediction of fetal macrosomia is poor. The aim of this study was to develop a reliable noninvasive prediction classifier of fetal macrosomia.

**Methods:**

A total of 3600 samples of routine noninvasive prenatal testing (NIPT) data at 12^+ 0^–27^+ 6^ weeks of gestation, which were subjected to low-coverage whole-genome sequencing of maternal plasma cell-free DNA (cfDNA), were collected from three independent hospitals. We identified set of genes with significant differential coverages by comparing the promoter profiling between macrosomia cases and controls. We selected genes to develop classifier for noninvasive predicting, by using support vector machine (SVM) and logistic regression models, respectively. The performance of each classifier was evaluated by area under the curve (AUC) analysis.

**Results:**

According to the available follow-up results, 162 fetal macrosomia pregnancies and 648 matched controls were included. A total of 1086 genes with significantly differential promoter profiling were found between pregnancies with macrosomia and controls (*p* < 0.05). With the AUC as a reference，the classifier based on SVM (C_MA-A2_) had the best performance, with an AUC of 0.8256 (95% CI: 0.7927–0.8586).

**Conclusions:**

Our study provides that assessing the risk of fetal macrosomia by whole-genome promoter nucleosome profiling of maternal plasma cfDNA based on low-coverage next-generation sequencing is feasible.

**Supplementary Information:**

The online version contains supplementary material available at 10.1186/s12884-022-05027-w.

## Background

Fetal macrosomia was defined as fetal weight beyond 4000 g or 4500 g, regardless of gestational age. The prevalence of fetal macrosomia has increased globally over the last several decades, and it occurred in approximately 8% of all pregnancies in 2016 [1]. Several studies reported that it is associated with an increased risk of the maternal and neonatal complications, including increased likelihoods of emergency cesarean section (CS), shoulder dystocia, brachial plexus injury, perinatal asphyxia and neonatal mortality [1, 2]. Furthermore, a large body of literature has reported associations between macrosomia and long-term health risks, such as adult obesity, diabetes, cardiovascular disease and cancer [3–8].

At present, the accuracy of antenatal estimation of fetal weight and prediction of macrosomia is poor. Both ultrasound biometry and clinical assessments, such as measuring fundal height, are commonly used methods to evaluate fetal intrauterine growth, but only with a sensitivity from 37 to 54% [9]. Moreover, those models could be used only during late pregnancy up to labor. Currently, the ability to block or delay the progress of macrosomia in the third trimester is limited; planned delivery by cesarean section is the most common clinical treatment to avoid further adverse influences on maternal and neonatal outcomes. Hence, the early diagnosis of fetal macrosomia might contribute to successful health monitoring during pregnancy and reduce the incidence of maternal and neonatal complications.

Cell-free DNA (cfDNA) in plasma is an intensively applied serological marker. In healthy people, cfDNA is released by apoptotic myeloid and lymphoid cells after enzymatic processing; in particular physiological conditions, such as pregnancy, 10% ~ 15% of cfDNA is derived from apoptotic placental trophoblasts, namely, cell-free fetal DNA (cffDNA) [10], which allows collection of fetal genetic information via noninvasive maternal blood tests. Currently, noninvasive prenatal testing (NIPT) is offered as a kind of screening test for common aneuploidies (e.g., trisomies 21, 18 and 13) in China. Additionally, cfDNA has also been used to screen for certain clinically significant microdeletions and single-gene disorders [11–14].

In several analyses, the average length of cfDNA fragements was 167 bp, a size which corresponds approximately to the DNA wrapped around a nucleosome (~ 147 bp) and a H1 linker fragment (~ 20 bp). Therefore, it has been accepcted that cfDNA is the fragment bounded to nucleosome [15–17]. Furthermore, evidence that cfDNA sequencing yields a genome-wide map of in vivo nucleosome occupancy and enables identification of its cell types of origin was recently reported [18]. In addition, a study of cfDNA analysis demonstrated that the genes with low-coverage in the region of ±1000 bp around the transcription start sites (TSSs) was active, which implied that low-coverage occurred in promoter regions along with increased gene expression [19]. In a previous study, our group proved that promoter profiling of cfDNA may be used as a biological biomarker for predicting pregnancy complications at early gestational [20]. Therefore, we hypothesized that cfDNA fragment distribution patterns can infer their tissues-of-origin, e.g. apoptotic placental trophoblasts, maternal myeloid and lymphoid cells, and the differential coverages in the region of ±1000 bp around the TSSs can be used to distinguish pregnant women with complications and healthy controls.

The aim of this study was to investigate the difference in the cfDNA nucleosome footprint profile between macrosomia and control pregnancies. Based on these differences, classifiers can be developed through machine learning for macrosomia prediction.

## Methods

### Study participants

The study was approved by the Internal Ethics Committee of Nanfang Hospital, Southern Medical University (NFEC-2017-049), and all women provided written informed consent for the use of their data in ongoing research before the blood draw. This was a nested case-control study. During the study period (Jan 1, 2016, and Aug 31, 2019), 3600 naturally conceived singleton pregnant women at 12^+ 0^ ~ 27^+ 6^ gestational weeks were recruited from three independent hospitals, namely, Nanfang Hospital of Southern Medical University (SMU), the Third Affiliated Hospital of Sun Yat-sen University (SYSU), and Cangzhou People’s Hospital. All plasma samples were subject to routine noninvasive prenatal testing.

### Inclusion and exclusion criteria

Based on pregnancy outcomes and neonatal birth weight, pregnancies were classified into the macrosomia and control groups. The exclusion criteria were as follows: 1) gestational age at blood collection (less than 12 weeks or more than 28 weeks); 2) maternal overweight or obesity (BMI over 25 before pregnancy); 3) multiple pregnancy; 4) singleton pregnancy with positive results on NIPT and ultrasound scans; 5) premature delivery; 6) birth weight below 2500 g and 7) lost in the follow-up. Pregnancies meeting any of these criteria were excluded. We identified macrosomia cases and controls by retrospectively analyzing the participant follow-up results, including pregnancy outcomes and neonatal birth weight. According to the gestational weeks at blood collection and the sex of the fetus, each macrosomia case was randomly matched to four selected control cases.

In total, 810 NIPT samples (162 macrosomia and 648 controls) were selected for further evaluation. Macrosomia was identified is cases with a birth weight beyond 4000 g.

### Sample processing and next-generation sequencing

Since pre-analytical factors can significantly affect cfDNA analysis [21, 22]. To guarantee that the samples from different hospitals would not be influenced by pre-analytical factors (e.g. storage temperature and time before processing), we have formulated protocols for quality control. The peripheral blood samples were collected from each participant in cfDNA BCT tubes, and centrifuged for 10 minutes at 1600×g to collect the plasma. And then, to remove the residual cellular fragments, plasma samples were centrifuged at 16,000×g for 10 minutes. All plasma samples were stored frozen at ≤ − 80 °C, and the cfDNA was extracted from those frozen samples by using the QIAamp DNA Blood Mini kit (Qiagen) by following the manufacturer’s instructions.

To construct DNA library, a total of 40.5 μL of extracted DNA was needed by means of TruSeq DNA Sample Prep reagents (Illumina, Paris). The DNA libraries were measured by using Qubit (Life Technocologies), and the integration of DNA were verified by using Agilent Bioanalyzer 2100 (Agilent Technologies). The purified libraries from twelve different individual samples were pooled, and massively parallel sequencing were performed on the Ion Proton sequencing platform (Life Technocologies) or the NextSeq500 sequencing platform (Illumina). The DNA sequencing was performed at a depth of 0.3× average coverage [23].

### Sequence analysis

After removal of sequencing reads with low quality, sequencing reads were aligned to the hg19 human reference genome using BWA-MEM [24], and PCR duplicated were removed SAMtools (ver. 1.2) [25]. Read counts of regions ranging from − 1000 bp to + 1000 bp around transcription start sites (TSS), defined as the primary transcription start site (pTSS), were calculated using BEDTools (ver. 2.17.0) and then normalized using the following formula:

1 $$\mathrm{Normalized}\ \mathrm{pTSS}=\frac{\mathrm{Reads}\ \mathrm{at}\ \mathrm{pTTS}}{\mathrm{Totally}\ \mathrm{mapped}\ \mathrm{reads}\times \mathrm{length}\ \mathrm{of}\ \mathrm{pTTS}\left(2\mathrm{kb}\right)}$$

### Prediction model construction and validation

**Fig. 1:**
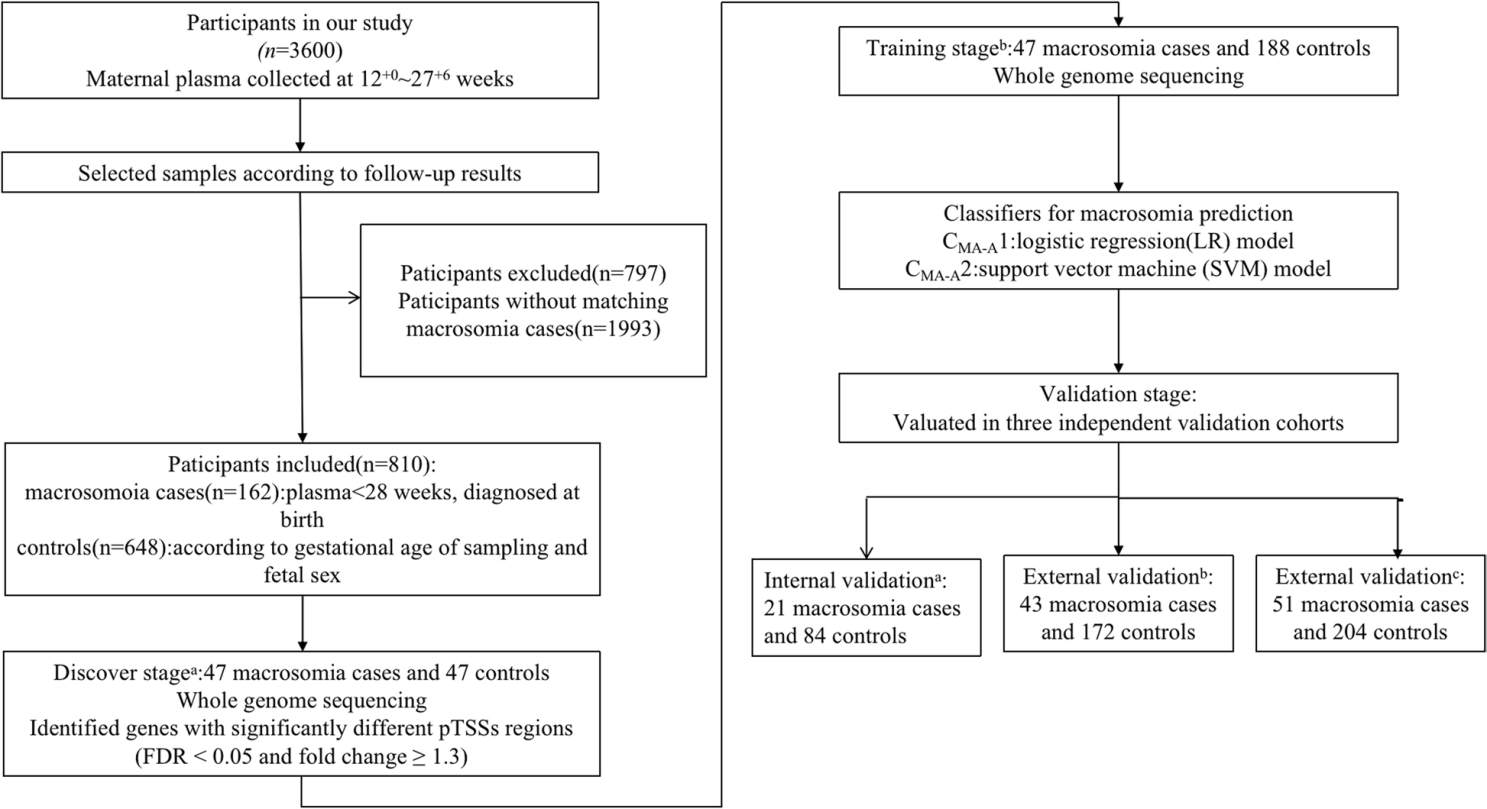
Study design flowchart for obtaining the macrosomia classifiers. a Samples collected from SMU. b Samples collected from SYSU. c Samples collected from Cangzhou People’s Hospital

To obtain effective classifiers for predicting pregnancies with fetal macrosomia, a three-stage workflow was designed, including exploration of genes with differential promoter profiling (discovery stage), construction of classifiers (training stage), and evaluation the performance of classifiers (validation stage) (Fig. 1).

At the discovery stage, we first sequenced cfDNA from maternal plasma samples collected from 47 macrosomia cases and 47 gestational age-matched controls, and the coverage at the pTSSs was compared between the two groups. *P*-values were then calculated using the Wilcoxon rank sum test and then adjusted to FDR using R software. pTSSs with fold change ≥1.3 and FDR ≤ 0.05 were considered significantly changed.

At the training stage, two machine learning models, including support vector machine (SVM) and logistic regression (LR), were used to develop promoter profiling-based classifiers to distinguish macrosomia cases from controls. To develop classifiers, a stepwise method was used to identify promoter combinations among genes showing differential coverage at the pTSSs. The robustness of the classifiers was assessed using leave-one-out cross-validation (LOOCV) [20, 23]. In brief, each subject in the training cohort was excluded from the training model in turn, with the remaining subjects all being submitted to train the model. The trained model was then used to predict the class (pregnancies with complications or controls) of the withheld subject. This procedure continued until all subjects in the training cohort were classified. The performance of each classifier was evaluated by using receiver operating characteristic (ROC) analysis, including area under curve (AUC), accuracy, sensitivity, and specificity. The classifier that achieved the maximum value of AUC in the training cohort, was considered to be the optimal classifier.

At the validation stage, for further evaluation, the efficiency of optimal prediction classifier was assessed in three validation cohorts, separately. The composition of internal cohort was samples collected from SMU, and cohorts composed of samples collected from SYSU and Cangzhou People’s Hospital were considered as external validation.

## Results

### Clinical characteristics

In total, we included 162 participants who developed macrosomia and 648 gestational age-matched controls (Fig. 1). These participants were divided into four cohorts: a training cohort (samples from Nanfang Hospital of SMU), an internal validation cohort (samples from Nanfang Hospital of SMU), and two external cohorts (samples from the Third Affiliated Hospital of SYSU and Cangzhou People’s Hospital). The clinical characteristics of the participants are summarized in Table 1. The gestational age and maternal age were not significant different in each cohort.

**Table 1:** Clinical characteristics of the study groups

	Training cohort			Internal cohort			External cohort 1			External cohort 2		
	MA	Con	P	MA	Con	P	MA	Con	P	MA	Con	P
Gestational age at sampling (weeks)	16.6 ± 3.5	16.4 ± 3.4	0.774	15.9 ± 3	15.9 ± 3.1	0.961	16.4 ± 3.4	16.4 ± 3.3	0.95	18.6 ± 3.8	18.8 ± 3.9	0.821
Maternal age (y)	33.3 ± 3.6	31.7 ± 5.0	0.176	35.7 ± 2.1	32.1 ± 5.1	0.027	34.0 ± 3.4	34.0 ± 3.3	0.953	30.6 ± 5.0	30.1 ± 4.9	0.537
Birth weight (g)	4147.9 ± 95.9	3292.2 ± 40.6	< 0.001	4291.6 ± 122.2	3311.1 ± 55.2	< 0.001	4228.9 ± 107.1	3289.3 ± 9.09	< 0.001	4137.1 ± 52.2	3218.2 ± 47.4	< 0.001

Data are the mean ± standard deviation

p = Mann-Whitney U test

*MA* Macrosomia

### Promoter and terminator profiling of cfDNA revealed macrosomia-associated patterns

**Fig. 2:**
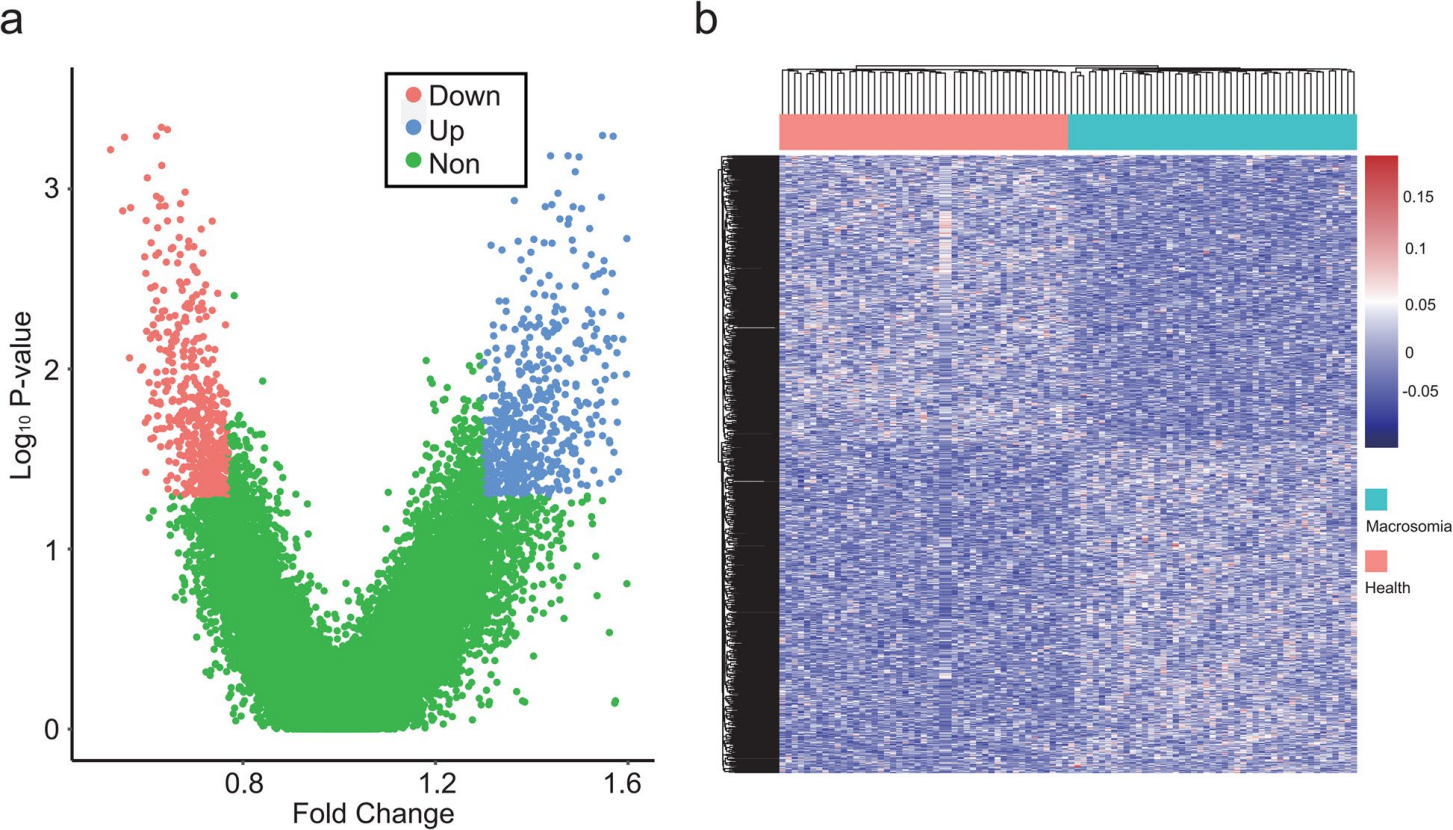
Gene transcripts with differential read coverages at primary transcription start sites (pTSSs). **a** Volcano plots of gene transcripts with differential read coverages at the TSSs, as detected using whole-genome sequencing for macrosomia. The blue, red and green blots indicate genes with upregulated, downregulated and no significant difference in promoter read depth coverage, respectively; **b** Heat map of the z-scores for promoters with differential read coverage using cfDNA-seq (FDR < 0.05). The two groups of women were separated using hierarchical clustering

To develop classifiers for predicting macrosomia, at the discovery stage, 47 pregnancies with macrosomia cases and 47 gestational age-matched controls were compared to identify gene transcripts with significantly different promoter and terminator coverage. A total of 1086 significantly different TSS regions (FDR < 0.05 and fold change ≥1.3) were found. The list of transcripts with significantly different promoter and terminator coverage is shown in supplementary materials (Supplemental Table). Hierarchical clustering analyses showed an obvious separation of pregnancy with macrosomia from controls (*P* < 0.05) (Fig. 2).

### Classifier development for macrosomia prediction

At the training stage, by the application of SVM and LR models, we constructed macrosomia classifiers that could differentiate the macrosomia cases from controls, and the performance of each macrosomia classifier was evaluated by ROC analysis. The unique 12-gene combination (C_MA-A1_), namely, *SMC3*, *MASTL*, *CREM*, *C1QTNF12*, *MLXIP*, *MAP3K9*, *IGSF6*, *APC2*, *GPM6A*, *TMEM128*, *NIPBL*, and *TMEM184A*, achieved the best performance with an AUC of 0.7793 (95% CI: 0.7094–0.8491) in the LR model. The accuracy of the C_MA-A1_ was 81.28%, with a sensitivity of 72.34% and specificity of 83.51%.

The unique 12-gene combination (C_MA-A2_), namely, *C10orf142*, *STX6*, *CORO1C*, *RCOR3*, *ITGB7*, *DUSP6*, *GSE1*, *RAB8A*, *KLC3*, *UBE2M*, *MCAT*, and *GLE1*, achieved the best performance with an AUC of 0.8298 (95% CI: 0.7675–0.8921) in the SVM model, and the accuracy of the C_MA-A2_ was 84.25%, with a sensitivity of 80.85% and specificity of 85.11%.

### Classifier validation for macrosomia prediction

**Fig. 3:**
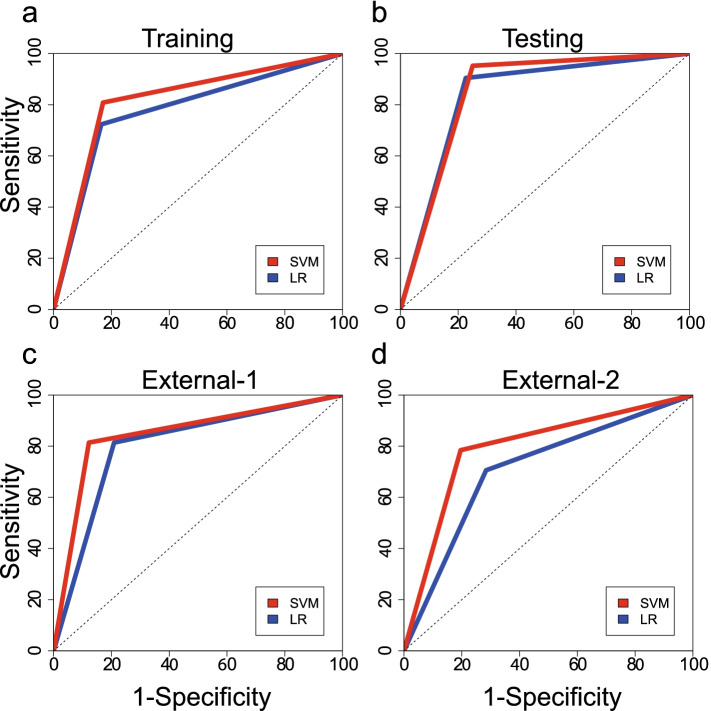
Receiver operating characteristic (ROC) curves. ROC curves showing the performance of SVM and LR models to predict macrosomia in different cohorts. **a** ROC curves for classifiers in the training cohort; **b** ROC curves for classifiers in the internal cohort; **c** ROC curves for classifiers in external cohort-1; **d** ROC curves for classifiers in external cohort-2

At the validation stage, to verify the efficiency of the optimal prediction classifier, the C_MA-A1_ and C_MA-A2_ were used in the internal cohort, 2 external cohorts and all subjects. The C_MA-A1_ had AUCs of 0.8393 (95% CI: 0.7608–0.9178), 0.8023 (0.7361–0.8686), 0.7108 (95% CI: 0.6404–0.7811) and 0.7716 (95% CI: 0.7352–0.808) in these four cohorts, respectively. The C_MA-A2_ had AUCs of 0.8512 (95% CI: 0.7853–0.9171), 0.8459 (95% CI: 0.7822–0.9097), 0.7941 (95% CI: 0.7309–0.8573) and 0.8256 (95% CI: 0.7927–0.8586) in these four cohorts, respectively (Table 2, Fig. 3). The C_MA-A2_ had achieved larger AUC and accuracy in each cohort, especially in all subjects (*P* < 0.05). According to the predictive performance of these two classifiers, we selected the C_MA-A2_ as optimal classifier for further discussion.

**Table 2:** Performance of the classifiers

Classifiers	LR				SVM				
Cohort	AUC (95% CI)	Acc	Sen	Spe	AUC (95% CI)	Acc	Sen	Spe	*P*-value
Training	0.7793 (0.7094–0.8491)	81.28%	72.34%	83.51%	0.8298 (0.7675–0.8921)	84.26%	80.85%	85.11%	0.111
Internal	0.8393 (0.7608–0.9178)	80.00%	90.48%	77.38%	0.8512 (0.7853–0.9171)	79.05%	95.24%	75.00%	0.655
External-1	0.8023 (0.7361–0.8686)	79.53%	81.40%	79.07%	0.8459 (0.7822–0.9097)	86.51%	81.40%	87.79%	0.219
External-2	0.7108 (0.6404–0.7811)	71.37%	70.59%	71.57%	0.7941 (0.7309–0.8573)	80.00%	78.43%	80.39%	0.005
All	0.7716 (0.7352–0.808)	77.53%	76.54%	77.78	0.8256 (0.7927–0.8586)	82.84%	82.10%	83.02%	< 0.001

*AUC* Area under the receiver operating characteristic curve, *95% CI* 95% confidence interval

### Performance of C_MA-A_ at different gestational weeks

To verify the the performance of C_MA-A2_ at different gestational age, we divided the samples into two groups according to the gestational ages at sampling. And the classifier was used to predict macrosomia in 455 samples (89 macrosomia cases) collected 17^+ 0^ gestational weeks and 355 samples (89 macrosomia cases) collected after 17^+ 0^ gestational weeks, respectively. C_MA-A2_ had AUCs of 0.8146 (95% CI: 0.7704–0.8588) and 0.8403 (0.7908–0.8898). To a certain degree, the accuracy of classifier increased with the progress of pregnancy. However, there was no evidence for a statistically significant difference in AUCs (*P* = o.449) (Supplemental Table 1). It suggested that the predictive performance of C_MA-A2_ was stable during 12^+ 0^ ~ 27^+ 6^ gestational weeks. Therefore, the prediction of macrosomia could start at 12^+ 0^ gestational weeks, as early as possible.

## Discussion

cfDNA-based NIPT has been successfully used in clinical practice as a kind of screening test for common aneuploidies, certain clinically significant microdeletions and single-gene disorders; moreover, since tumor cells can release shedding DNA fragments into the bloodstream, becoming a portion of the total cfDNA, cfDNA-based NIPT can be used as a “liquid biopsy” for cancer detection and monitoring.

In this study, we demonstrated that at 12^+ 0^ ~ 27^+ 6^ weeks of gestation, a method based on low-coverage whole-genome sequencing of cfDNA isolated from maternal circulating plasma was able to distinguish pregnancies of women who delivered an infant with macrosomia from those who delivered healthy neonates. We successfully developed- a classifier for predicting the risk of macrosomia based on maternal plasma cfDNA nucleosome profiling. The C_MA-A2_, based on SVM models, a combination of 12 genes, namely, *C10orf142*, *STX6*, *CORO1C*, *RCOR3*, *ITGB7*, *DUSP6*, *GSE1*, *RAB8A*, *KLC3*, *UBE2M*, *MCAT*, and *GLE1*, was the optimal gene combination to predict macrosomia, with the best performance and the highest prediction accuracy in 84.26% of the studied population (AUC, 0.8298, 95% CI: 0.7675–0.8921).

The classifier contained genes that may help us understand the etiology of fetal macrosomia. Previous studies have reported a close relationship between metabolic disorders and macrosomia, and these infants may also suffered from disordered glucose metabolism and obesity in later life [3, 26, 27]. C_MA-A2_ included *DUSP6* (dual specificity phosphatase 6), which is associated with lipid metabolism and glucose homeostasis [28, 29]. It has been reported that *CORO1C* (coronin 1C) were highly expressed in adipose tissues, and it might be critical genes in the development and prognosis of obesity [30]. And *ITGB7* (integrin subunit beta 7), is associated with Type 1 diabetes mellitus [31, 32]. Even though, the evidence of relationship between the remaining genes and macrosomia haven’t been reported yet. Considering the relevant studies about macrosomia is rare, especially the verification of gene function. It is necessary that further experiments are carried out to confirm the biological sense of C_MA-A2_.

Currently, fetal birth weight prediction is not a part of routine prenatal examination before the late period of pregnancy. There are not sufficient studies on how prenatal examinations can screen for fetuses at risk of macrosomia, especially in nondiabetic pregnant women. Currently, two-dimensional ultrasound is the most widely used prenatal technology for estimating fetal weight by measuring conventional parameters, including abdominal circumference (AC), biparietal diameter (BPD), and femur length (FL). Given the poor capacity of routine two-dimensional ultrasound for predicting macrosomia, a range of other techniques or models have been researched. In previous work, we developed a new birth weight prediction model based on the combination of two- and three-dimensional ultrasound that could improve the estimation accuracy of fetal birth weight [33]. However, these models can be used only during late pregnancy up to labor. Even though the fetal weight can be predicted through ultrasound during late pregnancy, it is unable to taking clinical intervention measures, like nutritional guidance, to prevent the fetal weight gain, effectively. To avoid the complications of vaginal delivery in the pregnancy of macrosomia, cesarean section (CS) can be chosen to terminate the pregnancy. However, as with any surgery, CS is associated with short- and long-term risk, and affect future pregnancies [34]. In our study, all maternal blood samples were collected in the first and second trimesters of pregnancy. This makes it possible to screen for fetal macrosomia at an early stage of gestation, and this method is earlier than other screening methods, would strengthen the management of pregnancy and would reduce the incidence of cesarean section.

Among the strengths of our study is the large sample size, comprising 162 cases of macrosomia and 648 controls. In addition, our classifier can be applied during the early period of pregnancy, when women have their routine NIPTs for chromosomal abnormality screening; it makes early intervention and management feasible. What’s more, there is no require for additional blood collection; it could be done as part of routine NIPTs without increasing the financial burden of women.

However, there are restrictions of this study. First, the retrospective design tends to engender overfitting, and the performance of the classifier is potentially overstated. Hence, before the classifier is applied to clinical practice, prospective studies that include pregnant women with singleton pregnancies in the first trimester are needed to further validate the prediction model. An additional restriction is that the construction of classifier merely included the coding genes. Previous studies have shown that the aberrant expression of miRNAs is related to macrosomia [35, 36]. However, to the best of our knowledge, the levels of macrosomia-related miRNAs in the plasma of pregnant women have not been investigated. Further analysis may provide novel insight into the roles that miRNAs have played in the intrauterine development of macrosomia. And a brand classifier model may achieve even more superior performance of predicting the risk of macrosomia prenatally, which is incorporate the TSSs of noncoding RNA.

## Conclusions

In summary, an important conclusion we can draw from our results is that a high-performance classifier was successfully developed for predicting the risk of macrosomia based on whole-genome sequencing of cell-free DNA, with potential for clinical implementation. It also might contribute to establishing new models for identifying and predicting other pregnancy-related diseases.

## Supplementary Information

**Additional file 1.**

**Additional file 2.**

## Data Availability

The datasets generated and analyzed during the current study are not publicly available due to the hospital policy but are available from the corresponding author on reasonable request.

## Abbreviations

NIPT: Noninvasive prenatal testing
cfDNA: Cell-free DNA
SVM: Support vector machine
AUC: Area under the curve
CS: Cesarean section
cffDNA: Cell-free fetal DNA
pTSS: Primary transcription start site
RPKM: Reads per kilobase per million mapped reads
LR: Logistic regression
LOOCV: Leave-one-out cross-validation
ROC: Receiver operating characteristic
AC: Abdominal circumference
BPD: Biparietal diameter
FL: Femur length
