# Supplementary material for: Performance of whole-genome promoter nucleosome profiling of maternal plasma cell-free DNA for prenatal noninvasive prediction of fetal macrosomia: a retrospective nested case-control study in mainland China
Source: BMC Pregnancy Childbirth. 2022 Sep 10;22:698. doi: 10.1186/s12884-022-05027-w (PMC9463826; doi:10.1186/s12884-022-05027-w)
Supplement: Supplementary file 2 — Additional file 2. [file 12884_2022_5027_MOESM2_ESM.docx]

**Supplemental Information**

**Supplemental information about C_MA-A1_**

The unique 12-gene combination (CMA-A1), namely, SMC3, MASTL, CREM, C1QTNF12, MLXIP, MAP3K9, IGSF6, APC2, GPM6A, TMEM128, NIPBL, and TMEM184A, achieved the best performance with an AUC of 0.7793 (95% CI: 0.7094-0.8491) in the LR model. And the probability of pregnancies with macrosomia was calculated by using a formula involving these 12 genes as follows:

| C_MA-A1_(logit[P=MA]) = $2.180+0.605\times SMC3-1.204\times MASTL+1.366\times CREM-1.295\times C1QTNF12-0.471\times MLXIP-0.811\times MAP3K9-1.284\times IGSF6-1.347\times APC2-0.504\times GPM6A+1.048\times TMEM128-0.057\times NIPBL-1.652\times TMEN184A$ |  |
| --- | --- |
|  |  |

**
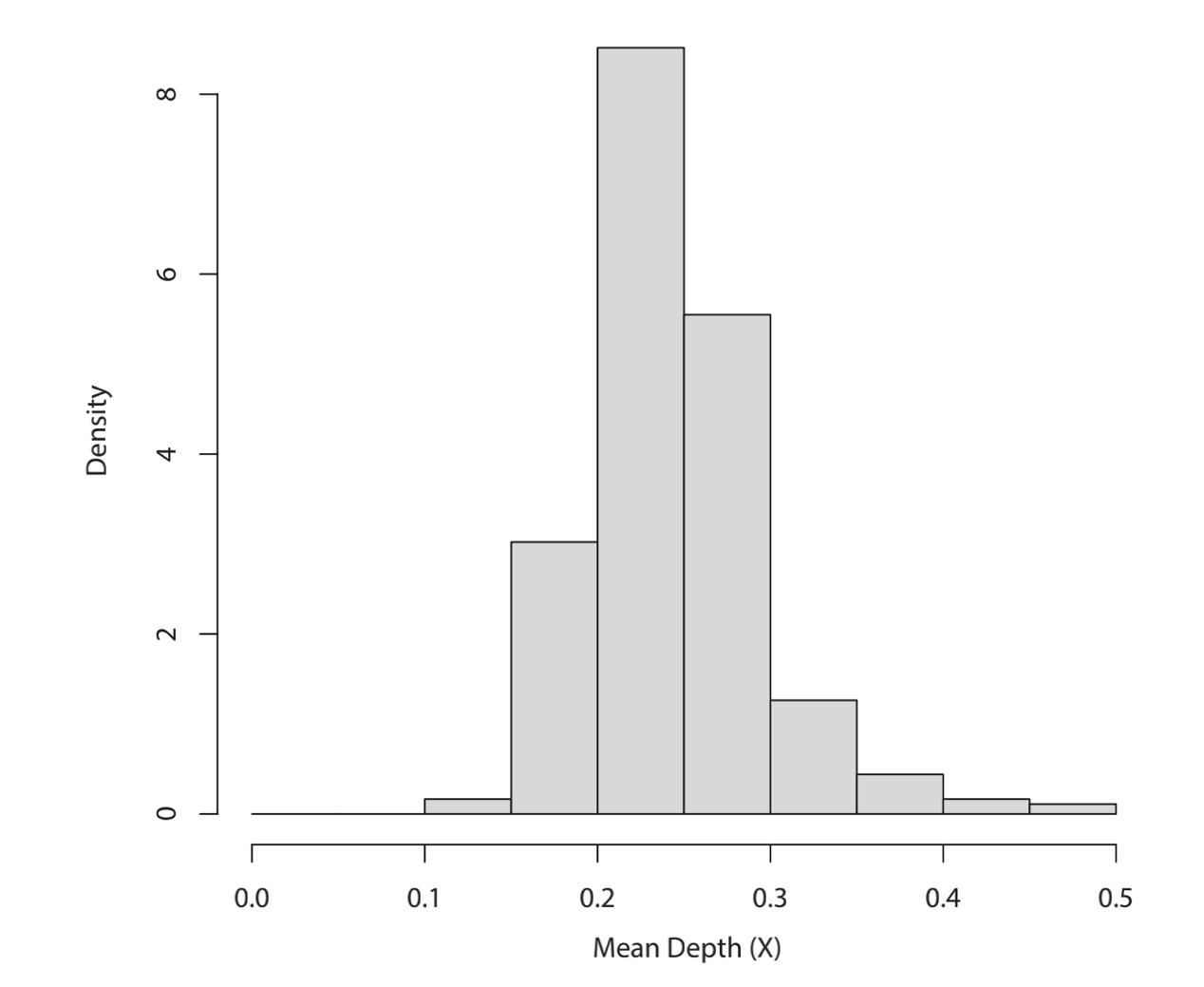
**

**Supplemental figure 1.** The density of sequencing data in mean depth

**Supplemental Table 1.** Performance of C_MA-A2_ in different gestational ages. GA, gestational age; MA, macrosomia.

|  | GA < 17^+0^ weeks | GA ≥ 17^+0^ weeks |
| --- | --- | --- |
| MA cases | 89 | 366 |
| Controls | 73 | 282 |
| AUC (95% CI) | 0.8146 (0.7704-0.8588) | 0.8403 (0.7908-0.8898) |
| P-value | 0.449 |  |
